# Characterization and Stability of the Antimony-Quercetin Complex

**DOI:** 10.15171/apb.2019.051

**Published:** 2019-08-01

**Authors:** Valcilaine Teixeira Barbosa, Janaína Barros de Menezes, Josué Carinhanha Caldas Santos, Maria Lysete de Assis Bastos, João Xavier de Araújo-Júnior, Ticiano Gomes do Nascimento, Irinaldo Diniz Basílio-Júnior, Luciano Aparecido Meireles Grillo, Camila Braga Dornelas

**Affiliations:** ^1^Departamento de Farmácia, Escola de Enfermagem e Farmácia, Universidade Federal de Alagoas.; ^2^Instituto de Química e Biotecnologia, Universidade Federal de Alagoas.; ^3^Departamento de Enfermagem, Escola de Enfermagem e Farmácia, Universidade Federal de Alagoas.

**Keywords:** Antimony, Binding sites, Flavonoids, Quercetin

## Abstract

***Purpose:*** Quercetin is a flavonoid known for its therapeutic properties and for forming complexes. Although the antimony-quercetin (SbQ) complex has been produced before, no previous exploration of its characteristics has been published in literature. Thus, this study aimed to characterize this complex, assess its stability and investigate its complexation site through its antibacterial activity.

***Methods:*** The SbQ complex was synthetized using Sb(III) potassium tartrate trihydrate and quercetin anhydrous (1:1) (v/v) as a solution and dried using three methods: rotaevaporation, lyophilization and spray drying. The material, in solution, was analyzed by UV-vis and fluorimetry; and, in the powder, by X-ray diffraction (XRD), both scanning electronic and fluorescence microscopy and infrared spectroscopy (FT-IR). Antimicrobial activity was evaluated via broth microdilution.

***Results:*** UV-vis exhibited a shoulder peak at 291 nm indicating metal chelation at C-ring of quercetin and confirmed 1:1 stoichiometry. Spectrofluorimetry showed an increase of intensity with the complex formation with an emission band (525 nm). After drying, XRD and SEM indicated loss of crystallinity and a difference in shape and size of the complex compared to its precursors. FT-IR suggested by a shift of frequency of the carbonyl group (1661 cm-1) that the quercetin bond to antimony by the C-3, followed by positions C-5 and C-4 carbonyl, which has been confirmed by MIC through the structure-activity relationship of the antibacterial activity of quercetin.

***Conclusion:*** These results provided a characterization of SbQ complex with the confirmation of its binding site, working as a guide for future studies involving this complex.

## Introduction

Quercetin is a flavonoid with biological properties, such as antioxidant, antibacterial and antitumor activity. This compound is characterized as a polyphenol with five hydroxyl groups in positions 3, 3’, 4’, 5 and 7 and a carbonyl in position 4.^1^ It is mainly found in plants, and is one of the compounds most widely present in the human diet.

Due to its polyhydroxylated chemical structure, quercetin easily forms complexes with chemical species having free orbitals which may be occupied.^2^ Studies on quercetin complexation with different metals have already been published, suggesting that the main bonding sites are the carbonyl at position 4, the hydroxyls at positions 3 or 5, and the o-phenolic group which includes the hydroxyls at positions 3’ and 4’.^3^

Moreover, it has been verified that the formation of compounds with metallic ions may change the properties of quercetin. Quercetin complexes with Cd(II), Cu(II), and Fe(II) have been assessed due to their effects on antioxidant capacity in comparison to free quercetin.^1,4,5^ In some cases, such as in the complex with Cu(II)^4^ and Fe(II)^5^ using DPPH^•^ there was an improvement of the antioxidant activity, however, in the case of complexes with Cd(II), using DPPH^•^ and ABTS^•+^, it has been noted that complexation had a negative effect on the antioxidant activity.^1^ The creation of complexes of quercetin with rare earth metals (La, Nd, Eu, Gd, Tb, Dy, Tm and Y) had the objective of increasing antitumor activityand, for complexes with La, Eu and Gd, cytotoxicity effects were better for cells of the Bel-7402 cell line, with a suppression ratio of 46.03, 63.32 and 45.30%, compared to a low suppression ratio for free quercetin (20.15%).^6^

Antimony-based products are used mainly for leishmaniasis chemotherapy, since leishmanicidal activity has been attributed to this semi-metal. Furthermore, studies with complexes involving antimony(III) and carbazones suggest an improvement in antimicrobial activity in comparison to free ligands.^7,8^ The formation of the quercetin-antimony complex has been explored previously for analytical purposes, both for flavonoid quantification and for water remediation treatment.^9,10^ However, there has been no previous assessment of the structural properties of this complex and of the influence of the complexation process in antibacterial activity. Additionally, no previous analysis exists for the use of this complex in solid form (powder), which could increase its stability, enabling it to be stored.

For the reasons mentioned above, this study aims to characterize the antimony(III)-quercetin (SbQ) complex in solution and in powder form through different drying procedures, as well as to investigate its complexation site through its influence on antibacterial activity.

## Materials and Methods

### Material

Antimony(III) potassium tartrate trihydrate from Vetec (Duque de Caxias, RJ, Brazil) and anhydrous quercetin from Sigma-Aldrich (St. Louis, MO, USA) for SbQ complex. For antibacterial assay, it was used brain heart infusion (BHI), rutin hydrate both Sigma-Aldrich Co. (Saint Louis, MO, USA), triphenyltetrazolium chloride (TTC) from Inlab (São Paulo, SP, Brazil) and NaCl from Synth (Diadema, SP, Brazil). Cell lines of bacteria* Staphylococcus aureus* (CCCD-S007) and *Escherichia coli* (ATCC-25922). The solvents used were ultrapure water from Merck (Darmstadt, Germany), methanol from Dinâmica (Indaiatuba, SP, Brazil) and dimethylsulphoxide (DMSO) from Synth (Diadema, SP, Brazil).

### Synthesis of the antimony-quercetin complex in solution (SbQ-S)

The volumes of 50 mL of antimony(III) potassium tartrate (3 mM) aqueous solution and 50 mL of quercetin methanol solution (0.66 mM) were mixed directly without mechanical shaking, having as a result immediate formation of the SbQ complex in solution (SbQ-S).^9^

### Characterization of the SbQ-S complex

Formation of the SbQ-S was evidenced by UV-vis spectrophotometry (Micronal AJX-6100PC spectrophotometer, SP, Brazil) and molecular spectrofluorimetry (Shimadzu, RF-5301PC spectrofluorimeter, Tokyo, Japan) with excitation at 370 nm.^9^ Job’s method was used to determine the stoichiometry.^11^

### SbQ complex drying process

SbQ-S went through three different drying processes using the following techniques: rotaevaporation, lyophilization and spray drying. The resulting powders compounds were designated as SbQ-R, SbQ-L and SbQ-SD, respectively. It is important to mention that before obtention of SbQ-L and SbQ-SD, there were previously rotaevaporation process at 60°C for methanol removal and then lyophilization and spray drying, respectively.

In the obtention of SBQ-R was used an IKA^®^ RV10 rotary evaporator at 80°C (Staufen, Germany). For SBQ-L, the solution was placed in a freezer for 48h prior to the drying procedure, in which was used Terroni^®^ LD1500 lyophilizer (São Carlos, Brazil). Lastly, for spray drying was utilized a Büchi B-290 mini-spray dryer (New Castle, USA) with an inlet temperature of 200°C and outlet temperature varying between 75% and 85°C, 33% pump rate and 85% aspirator rate. All procedures were performed and all products were stored away from light.

### Characterization of the complexes in powder form (SbQ-R, SbQ-L and SbQ-SD)

Product characterization in powder form used: powder X-ray diffraction (XRD) in a Shimadzu XRD-6000 (Kyoto, Japan); scanning electron microscopy (SEM) in a Shimadzu SSX-550 microscope (Kyoto, Japan); and Fourier transform infrared spectroscopy (FT-IR) in a Thermo Scientific Smart OMNI-Sampler Nicolet iS10 FT-IR Spectrometer (Massachusetts, USA). Compounds were also restituted and analyzed by FT-IR, transillumination in Gel logic 200 Kodak (New York, USA) and fluorescence microscopy in a ZEISS Observer Z.1 Apo Tome microscope (Oberkochen, Germany).

The XRD used CuKα with 30 kV voltage and 30 mA current, a Ni filter and data collected in a 2θ range between 3-40 degrees. For SEM, samples were previously coated in gold with a metallizer with a 10 mA current for 5 minutes. FT-IR used attenuated total reflectance (ATR) with a wavenumber range between 4000 and 750 cm^-1^. For fluorescence microscopy, samples were fixed in slides using ethanol. Transillumination used a wavelength of 306 nm, and powders were restituted in their respective solvents. Because of the maintenance of powders’ characteristics, fluorescence microscopy analyses and FT-IR of restituted powders SbQ-R was used as a model.

### Assessment of antibacterial activity

A broth microdilution assay was used to assess antibacterial activity following the protocol defined previously.^12^ Firstly, all materials were sterilized and BHI was used as a culture medium. Analyses were carried out in sterile polystyrene plates (96 wells), in triplicate, using *Staphylococcus aureus* and *Escherichia coli* suspension with 0.9% saline solution based on 0.5 McFarland scale. A stock solution of 1000 µg mL^-1^ of all samples were done using DMSO 1% (v/v). The treatments were as followed: positive control (PC)-BHI and bacterial solution; negative control (NC)-BHI and DMSO; sterility control (SC)-BHI. The plates were incubated at 37°C for 24 hours, and then it was used TTC 5% (m/v) as a bacterial growth indicator. Minimum Inhibitory Concentration (MIC) was evaluated and values below 500 µg mL^-1^ were used as reference for acceptable bacterial inhibition.^13^ SbQ-S was not analyzed due to it containing methanol, which could produce false positives. Rutin was tested as an inhibitory control.

### Statistical analysis

The quantitative results, when necessary, were expressed as mean ± SD of three independent experiments (n = 3), where SD is equal to the standard deviation.

## Results and Discussion

### Characterization of SbQ complex

**Figure 1:**
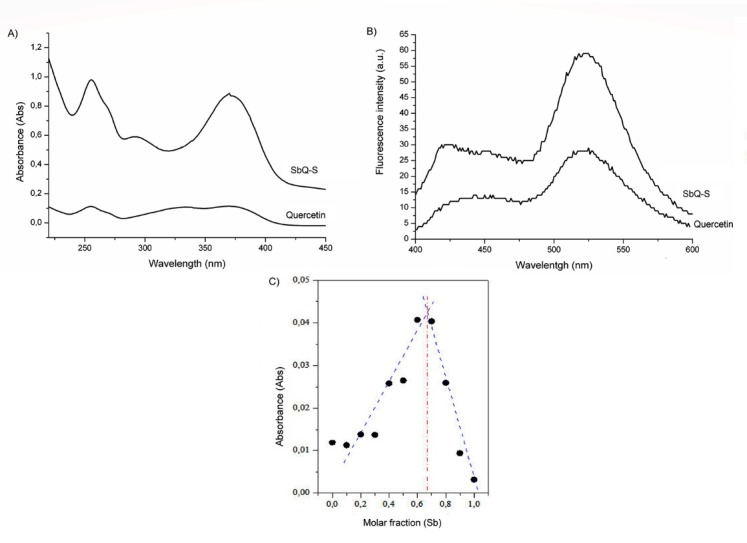
A) UV-vis spectra of quercetin (5.3 µM), SbQ-S complex (0.04 mg mL^-1^); B) Fluorescence spectra of the SbQ-S complex in relation to quercetin (both in 0.04 mg mL^-1^) and C) Job continuous variation plot for determination of the complex’s proportion. In all system was used ethanol:water medium.

The SbQ-S complex was obtained from the room temperature stirring quercetin and antimony(III) potassium tartrate in methanol:water (1:1) with a final pH 6.0. Initially the products were characterized by UV–vis (Figure 1A), in which quercetin showed two absorption bands. The band I refers to ring B and is located in the wavelength of 300-400 nm, while band II, located in the UV range of 240-300 nm, is related to ring A.^14^ The present work demonstrated these two bands in 369 and 255 nm are of quercetin. Antimony(III) did not present absorption at the range analyzed (data not shown).

Related to SbQ-S, a change of color from yellow to orange could be noted and represents the complex formation.^9^ There was no substantial shift comparing the absorption of free quercetin and SbQ-S as was expected, however, there was a shoulder peak at 291 nm, which indicates a participation of metal chelation at C-ring of quercetin.^15^ Literature has found that pH has influence on the capacity and stability of quercetin complexation, where also no shifting results were obtained in an acidic medium for quercetin-cadmium complex.^1^ It happens due to the predominance of the undissociated form of flavonoids in acidic medium, thus the complex formation become difficult as much as pH has tendency to acid medium.^2^

With respect to the molecular fluorescence spectrums (Figure 1B), free quercetin and SbQ-S displayed a weak peak at around 430 nm and a strong emission at 525 nm and it was noted an increase of intensity with the complex formation. The same was seen in complexes using quercetin and rare earth metals.^6^ It was expected to see quercetin fluorescence knowing that, in pH 6.0, it is presented in a neutral form with at least two tautomers, in which at least one presents fluorescence. There has been seen in literature that a weak band at 430 nm and strong emission at 540 nm correspond, respectively, to the excited state formed upon intramolecular proton transfer (ESPT) and the local excited state (LE) of the 3OH intramolecular hydrogen bonded tautomer, which corroborated our results.^16^

After confirming the complex formation, it was analyzed the probable stoichiometry of the complex, Job’s method suggested a formation of a ternary complex, in which tartrate was still linked to the SbQ-S complex. Based on the plot obtained (Figure 1C), the antimony: quercetin proportion found in the analyzed conditions (0.64 for antimony, 0.36 for quercetin) could be identified as 2:1. However, given the presence of two atoms of antimony in the antimony(III) potassium tartrate precursor, the stoichiometry must be corrected to 1:1.

**Figure 2:**
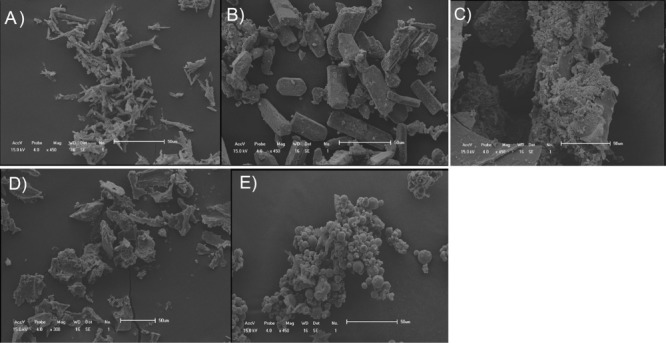
Microstructure of the compounds through SEM in: A) quercetin; B) antimony(III) potassium tartrate; C) SbQ-L; D) SbQ-R; E) SbQ-SD.

When the powders were observed by scanning electron microscopy (Figure 2) images show that the precursors, such as quercetin, has the form of thin sticks with lengths below 30 nm (Figure 2a), while antimony (III) potassium tartrate, was structured in apparently rectangular blocks larger than 50 nm (Figure 2b). The complexes formed varied structures: SbQ-R (Figure 2c) formed block clusters with lengths larger than 100 nm; SbQ-L (Figure 2d) had a heterogeneous form and approximate size under 30 nm; and SbQ-SD (Figure 2e) formed clusters of differently sized spheres, always smaller than 25 nm.

**Figure 3:**
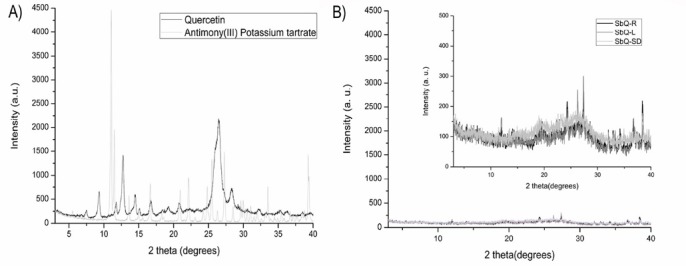
Diffractogram of A) Precursors (antimony(III) potassium tartrate and quercetin); B) Dried SbQ complexes (SbQ-R, SbQ-L, SbQ-SD) with a magnification inside for a better visualization.

The XRD results allowed visualization of crystallinity differences between the precursor materials and the complexes (Figure 3). The precursors, antimony (III) potassium tartrate and quercetin, showed well-demarcated signs with high intensity, a result coherent for crystalline structures. The crystalline planes of quercetin in this study suggest a mixture of crystalline forms of QCTa and QCTb samples, named and described in the literature.^17^ This profile was lost after drying, regardless of the technique used, confirming formation of a new compounds with amorphous characteristics.

**Figure 4:**
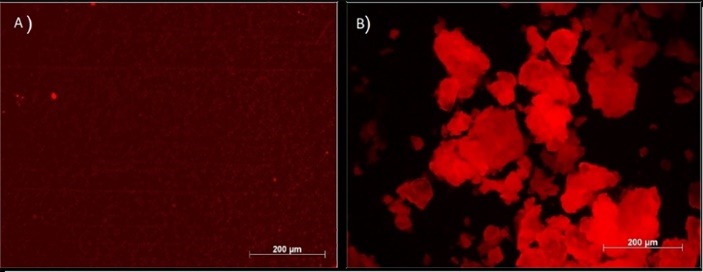
Micrography through fluorescence microscopy for: A) quercetin and B) SbQ-R.

**Figure 5:**
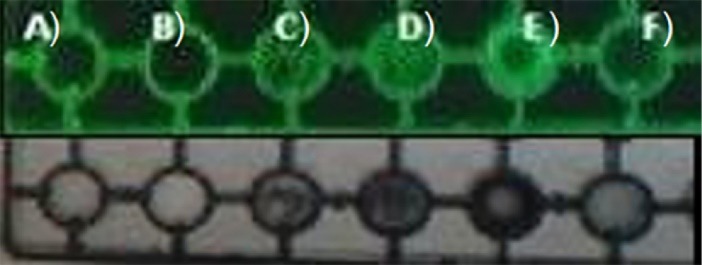
Samples after transillumination (306 nm): A) quercetin; B) antimony (III) potassium tartrate; C) SbQ-S; D) SbQ-R; E) SbQ-L and F) Sb-SD.

Fluorescence microscopy (Figure 4) and transillumination (Figure 5) techniques show maintenance of the fluorescence, which characterizes the complex, except in the SbQ-SD system, which had lower intensity in transillumination than the other systems. In general, however, it is possible to infer that the drying procedures had little influence on this important characteristic of the complexes.

### Investigation of the complexation site

**Figure 6:**
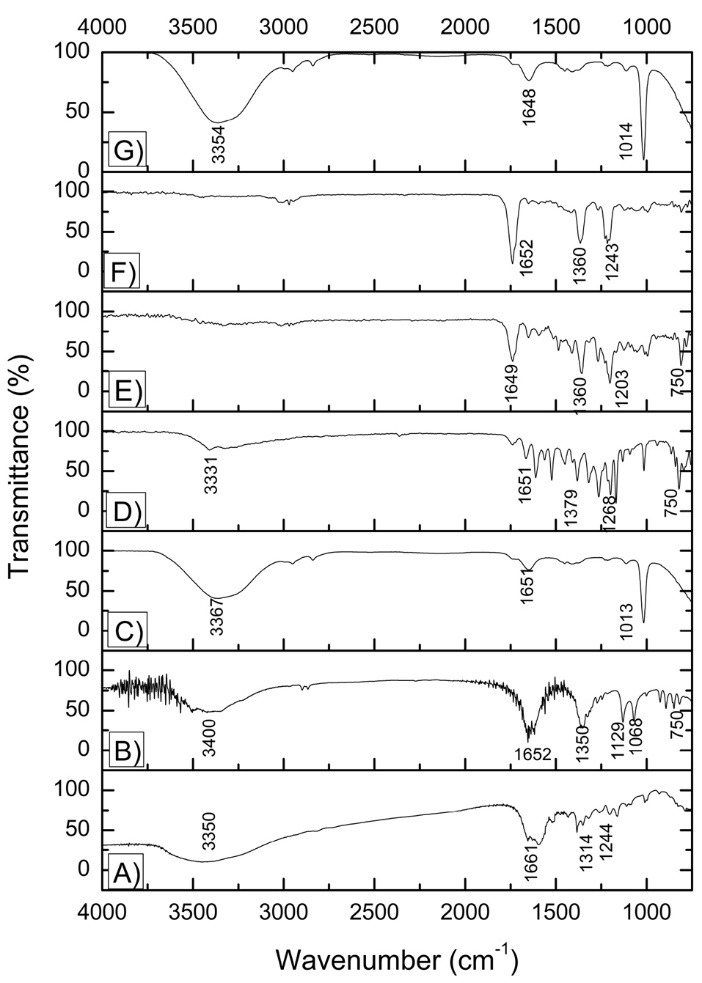
FT-IR Spectrums. A) quercetin; B) antimony (III) potassium tartrate; C) SbQ-S; D) SbQ-R; E) SbQ-L; F) SbQ-SD; and G) SbQ-R (restituted).

Compiled FT-IR data is shown in Figure 6. This technique allowed observation of the maintenance of the complex’s characteristics, being essential for the study of the region of the antimony (III)-quercetin interaction. The quercetin spectrum (Figure 6a) allows visualization of its main bands, with one weak band at 3350 cm^-1^ related to the axial deformation of the OH bond and intramolecular hydrogen bonds from free hydroxyl groups; ν(C=O) stretching at 1661 cm^-1^ related to the carbonyl; a band at 1314 related to bending C-OH phenol and a band at 1244 cm^-1^, related to the flexion of the C-O-C by stretching of the C-O-C.^18^

For antimony(III) potassium tartrate (Figure 6b), a region between 3600 and 2250 cm^-1^ is observed that corresponds to the (ʋ)OH stretching related to adsorbed water, as well as the following bonds: COO- from carboxylic acid (1650 and 1350 cm^-1^) and C-O (1150-1085 cm^-1^) from alcohol.^19^ Bands 747 and 665 cm^-1^ correspond to the Sb-O bonds, since vibrations corresponding to metal-O bonds occur at this range.^20^

Comparing precursors with the obtained complexes, firstly it was possible to observe a reduction and even a disappearance of hydroxyl groups when dried SbQ complexes are formed. It is known that ATR technique used for FT-IR analysis that does not work well with weakly absorbing substances, what could be the case of the new compounds produced as it was seen a diminution of intensity of hydroxyl bands in a study of Cu-Quercetin complexes.^15^

For the SbQ-S complex (Figure 6c), a reduction in the frequency of the carbonyl group characteristic of quercetin was verified prior to the formation of the complex, from 1661 to 1651 cm^-1^. This was also observed for the dried complexes, SbQ-R, SbQ-L, and SbQ-SD (Figures 6d-f), which showed frequency reductions to 1651, 1649 and 1652 cm^-1^, respectively, and for restituted SbQ-R (Figure 6g), with frequency reduction to 1648 cm^-1^. This reduction suggests coordination of the carbonyl oxygen with the metallic (III) ion and of the 3-OH and/or 5-OH groups from the ligand after deprotonation to form the metal-O bonds in the complexes^6^ corroborating with UV-Vis results which indicated binding at C-ring. As the 3-hydroxyl group is the most acid, this is likely the first chelation site, since there is a higher electron delocalization in the 3-OH group and, after, the second site would be the 5-OH group. This facilitates delocalization of the π electrons, followed by complexation in the carbonyl at position 4, which was also found for copper-quercetin complexes.^15,17^

Therefore, in order to confirm the first binding point in this complexation, it has been observed in the literature that quercetin position C-3 is essential for its antibacterial activity justified by its capacity of membrane interaction^21^ and inhibition of DNA isomerization catalyzed by bacterial DNA gyrase.^22^ However, this activity is compromised by the presence of sugar in position C-3, since quercetin presents weak inhibition against bacterial DNA-gyrase in its glycosylated form, as is the case for rutin.^23^ In a study analyzing the formation of quercetin complexes with Mn(II), Co(II), Cd(II) and Hg(II) an increase in the antibacterial property of the complexes in comparison to free quercetin has been observed,^24^ however, the binding points found by these authors were the hydroxyls in the *o-*phenolic ring B, therefore not affecting position C-3.

Thus, it can be concluded that a reduction or inhibition of the antimicrobial biological property of quercetin would be expected if there were a binding in position C-3. An assessment of the antibacterial activity of quercetin, antimony (III) potassium tartrate and the complexes (SbQ-R, SbQ-L and SbQ-SD) was then carried out, with rutin used as a negative control (Table 1).

**Table 1:** Minimum inhibitory concentration (MIC) values found based on samples analyzed in triplicate (Quantitative results were expressed as mean ± SD)

**Bacterial strains**	**MIC (µg mL** ^-1^ **)**					
	**Que**	**Sb**	**SbQ-R**	**SbQ-L**	**SbQ-SD**	**Rutin**
*S. aureus* (G +)^*^	15.6 ± 0.9	31.2 ± 1.4	NI^***^	NI	NI	NI
*E. coli* (G-)^**^	250 ± 5.4	125 ± 4.8	NI	NI	NI	NI

^*^G+ = Gram positive bacteria; ^**^G- = Gram negative bacteria; ^***^NI = no inhibition (> 500 µg mL^-1^).

The MIC for *Staphylococcus aureus* (Gram +) and *Escherichia coli* (Gram -) were 15.6 and 250 µg mL^-1^, respectively, for quercetin, and 31.2 and 125 µg mL^-1^, respectively, for antimony(III) potassium tartrate. Rutin was inactive for both strains. All complexes showed the same behavior, displaying no significant activity; that is, the complexes were not able to inhibit the strains at concentrations lower than 500 µg mL^-1^. This result corroborates the thesis that SbQ complexation occurs in the carbonyl located at position 4 with involvement of the hydroxyl located at position C-3, which is the likely active site of the flavonoid for this activity, suggesting a so-called keto-enol chelation.

This phenomenon happens due to a process known as tautomerism.^25^ In quercetin, the reactivity of the 3-OH group is generated by the presence of the 2,3 bond of the C ring where, in this case, removal of the H from the 3-OH group may be induced both by removal of a H atom from the enolic form of quercetin, which induces the C-2 atom, and by removal of a H atom from the C-2 atom of the keto form of quercetin, which may as a consequence form a keto-enol tautomerism through the double bond at position 2,3.^26^

**Figure 7:**
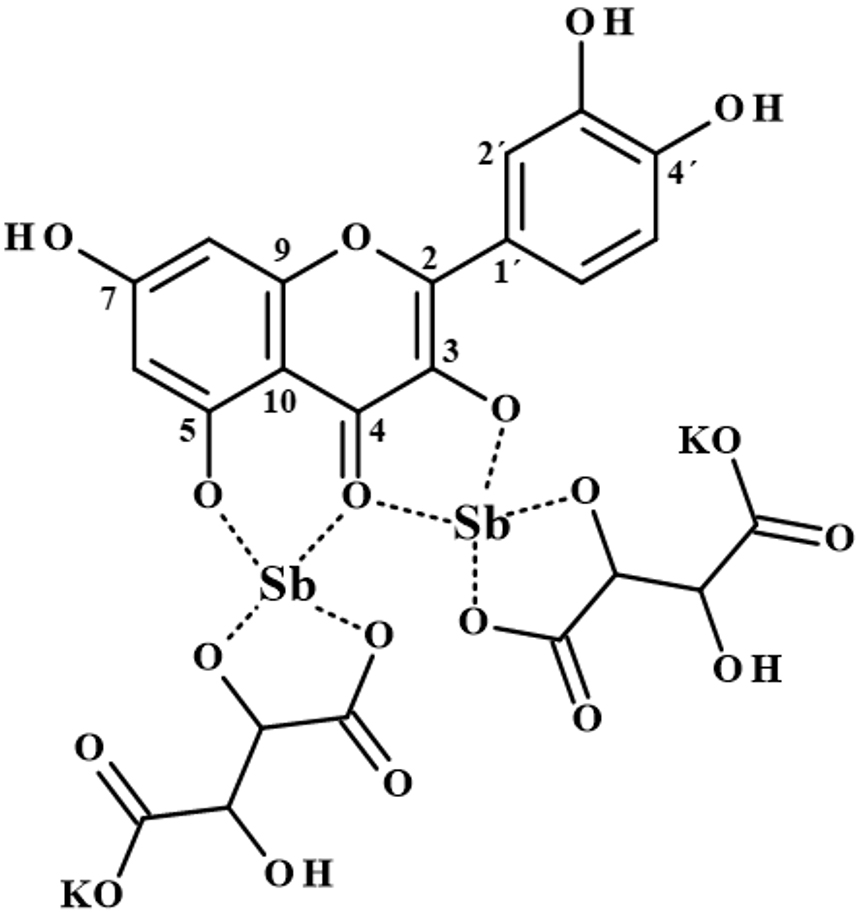
Representation of the proposed SbQ complex structure.

Considering that mild conditions were used in this work (ambient temperature without addition of base), and that antimony potassium tartrate was used as the source of Sb, the formation of possible Sb oxides during the synthesis process is limited, since more drastic reaction conditions are required.^19,27,28^ Tong et al also performed the synthesis of the SbQ complex but using SbCl_3_ as antimonium source and applying different experimental procedures to that used in this work and not observed the oxides formation.^29^ Therefore, based on the information obtained, Figure 7 provides a schematic representation of the structure of the SbQ complex formed based on our reactional conditions.

## Conclusion

Thus, the SbQ complex has been obtained successfully both in solution and in powder form using different drying processes. FT-IR was particularly important in providing data indicating the complex’s binding site. However, analysis of the structure-activity relationship through antibacterial activity tests was also an important tool to corroborate the site of antimony-quercetin interaction, confirming the bond of Sb at position C-3 through the complex’s inactivity in relation to its precursors, as occurs in rutin (which has this position occupied), and followed by positions C-5 and C-4 carbonyl. Therefore, this study provides an important contribution regarding the characterization of the antimony-quercetin complex, which may help in future studies assessing these compounds.

## Ethical Issues

Not applicable.

## Conflict of Interest

The authors declare that they have no conflict of interest.

## Acknowledgments

This work was partially supported by FACEPE/CETENE (# Grant APQ-1456-3.03/15), CNPq and FINEP (Brazilian Research Agencies) as well as FAPEAL (Alagoas State Research Agency). This study was financed in part by the Coordenação de Aperfeiçoamento de Pessoal de Nível Superior - Brazil (CAPES) - Finance Code 001.
